# Recognizing Frailty Through Pictures: Turkish Validation of the Pictorial Fit–Frail Scale in Older Adults

**DOI:** 10.3390/medicina62071330

**Published:** 2026-07-10

**Authors:** Serap Boz, Ayse Dikmeer, Rana Tuna Dogrul, Kubra Erdogan, Gokberk Gozukan, Arzu Nevin Dagdemir, Busragul Yılmaz, Fatma Kaplan Efe, Rıdvan Erten, Ertugrul Demirel, Hande Selvi Oztorun, Gunes Eken, Kamile Silay

**Affiliations:** 1 Department of Geriatrics, Ankara Bilkent City Hospital, Ankara 06800, Türkiyerana_tuna@hotmail.com (R.T.D.); kubraacmn@hotmail.com (K.E.); gokberkgozukan@gmail.com (G.G.); anevincoskun@gmail.com (A.N.D.); drbusragulyilmaz@gmail.com (B.Y.); drefe.106@gmail.com (F.K.E.); dr.ridvanerten@gmail.com (R.E.); drertugruldemirel@gmail.com (E.D.); drhandeslv@hotmail.com (H.S.O.); drgunesarik@gmail.com (G.E.); kamilesilay@hotmail.com (K.S.); 2 Division of Geriatrics, Department of Internal Medicine, Faculty of Medicine, Ankara Yıldırım Beyazıt University, Ankara 06010, Türkiye; 3 Geriatrics Clinic, Ankara Bilkent City Hospital, Faculty of Medicine, Health Science University, Ankara 06200, Türkiye

**Keywords:** pictorial fit–frail scale, frailty, aging, Turkish validation, reliability, validity

## Abstract

*Background and Objectives*: This study aimed to translate and culturally adapt the Pictorial Fit–Frail Scale (PFFS) into Turkish and to evaluate its validity and reliability in older adults. *Materials and Methods*: The study included 156 community-dwelling older adults aged ≥ 65 years. Frailty status was determined using the Clinical Frailty Scale (CFS), and participants with a CFS ≥ 4 were classified as frail. All participants underwent the PFFS, Katz ADL, Lawton–Brody IADL, Short-Form Geriatric Depression Scale (GDS), Mini Nutritional Assessment—Short Form (MNA-SF), Standardized Mini-Mental State Examination (SMMSE), and Charlson Comorbidity Index (CCI). The reliability of the PFFS was assessed using internal consistency (Cronbach α), inter-rater reliability, and test–retest reliability (ICC); its construct validity was assessed using correlation analysis with the CFS. Furthermore, the diagnostic performance of PFFS in identifying frailty was analyzed using the ROC curve. *Results*: Of the 156 individuals participating in the study, 63.5% were women, and 89 individuals (57.1%) were frail. The Turkish version of PFFS showed high internal consistency (Cronbach α = 0.838). Inter-rater and test–retest reliability were found to be ICC = 0.995 and 0.996, respectively. A strong and positive correlation was found between PFFS and CFS scores (Spearman r = 0.760, *p* < 0.001). ROC analysis showed that PFFS had good performance in distinguishing frailty (AUC = 0.851, optimal cut-off ≥ 14; sensitivity = 69.7%, specificity = 83.6%). Frail individuals were older, had higher comorbidities, and showed greater dependence in activities of daily living. *Conclusions*: The Turkish version of the PFFS appears to be a reliable, valid, rapid, and multidimensional tool for assessing frailty in older adults. The PFFS can contribute to the effective assessment of frailty in clinical practice and research.

## 1. Introduction

The global population is aging rapidly, making frailty an increasingly important public health challenge. Frailty is common among older adults living in the community, although reported prevalence rates differ substantially across studies. Current evidence suggests that frailty affects approximately 4–17% of adults aged 65 years and older, whereas prefrailty has been reported in 19–53% of this population. Such variability primarily reflects differences in the operational definitions of frailty, assessment methods, and the demographic and clinical characteristics of the study populations [1]. Frailty is a multidimensional geriatric syndrome characterized by reduced physiological reserve and increased vulnerability to stressors, leading to adverse outcomes such as falls, disability, hospitalization, cognitive decline, and mortality [2,3,4].

Age-related frailty is the result of cumulative cellular damage arising from various causes throughout an individual’s life; typical aging leads to a loss of homeostatic reserves in physiological systems. However, despite this loss of reserves, many individuals continue to function well as they age. Any stress or damage to these physiological reserves can lead to a deterioration in an older adult’s condition and, consequently, an increase in frailty. Patients can be classified as healthy, prefrail, or frail, depending on the degree of physiological and functional decline [5]. Early identification of frailty is essential because frailty is a dynamic and potentially reversible condition, particularly during the prefrail stage. Timely screening enables the implementation of interventions such as exercise, nutritional support, medication review, and comprehensive geriatric assessment, which may delay or even reverse the progression of frailty and improve health outcomes [6].

Although frailty is widely recognized as a clinically important syndrome, its assessment remains challenging because no universally accepted definition or gold-standard assessment tool currently exists. This lack of consensus reflects the complex etiology of frailty, the use of different conceptual models, and the difficulty of distinguishing frailty from normal aging and disability [7]. Although numerous tools have been developed to assess frailty, there is still no consensus in the literature regarding a standard test [8,9]. Commonly used assessment methods include the gait speed test, the Clinical Frailty Scale (CFS), the physical frailty phenotype, the frailty index, and the Short Physical Performance Battery. The duration of these tests can range from less than 10 min for simple methods to several hours for methods requiring stepwise assessment or specialized equipment [10].

Despite the availability of numerous frailty assessment tools, there is still no consensus regarding the optimal instrument for use across different clinical settings [11]. A recent scoping review identified 89 different frailty measures, highlighting the substantial heterogeneity in frailty assessment. Many commonly used instruments have important limitations. Performance-based tools, such as the frailty phenotype and the Edmonton Frailty Scale, require assessments of gait speed or grip strength, which may be impractical for severely frail individuals. Other instruments, including the FRAIL Scale, rely on a limited number of symptoms and may overlook valuable information provided by patients or caregivers. In addition, questionnaires such as the Tilburg and Groningen Frailty Indicators depend on verbal communication, limiting their applicability in individuals with dementia, communication difficulties, low health literacy, or language barriers. These limitations highlight the need for a simple, comprehensive, and user-friendly frailty assessment tool that can be applied across diverse clinical settings without requiring specialized geriatric expertise [12].

In this context, visual-based scales represent an important alternative to language-based tools. Visual scales have long been widely used, particularly in pain assessment, and facilitate understanding in individuals with communication difficulties [13]. Furthermore, most frailty measurement tools assess only the patient’s or only the clinician’s perspective, and the patient’s own perception can often be inconsistent with the assessments of caregivers or healthcare professionals. Therefore, it is important that an ideal frailty tool considers the views of all stakeholders and can be applied without requiring geriatric expertise [12]. Developed based on these needs, the Pictorial Fit–Frail Scale (PFFS) is a measure that can be completed by patients, caregivers, or healthcare professionals and assesses frailty across 14 domains using visual representations [14]. In clinical practice, it has been shown to take less than 2 min to complete by a healthcare professional and less than 5 min to complete independently or by a proxy. Furthermore, in a study conducted on an outpatient geriatric population with low health literacy and high rates of cognitive impairment, the PFFS was found to be both valid and feasible [15].

Therefore, the aim of this study was to translate and culturally adapt the Pictorial Fit–Frail Scale into Turkish and to evaluate its validity and reliability in older adults. Establishing a valid and reliable Turkish version of the PFFS may facilitate efficient frailty screening in clinical practice, support early identification of frailty, and contribute to the implementation of appropriate interventions aimed at improving quality of life and reducing functional dependence among older adults.

## 2. Materials and Methods

### 2.1. Study Design and Participants

This cross-sectional validation study included community-dwelling adults aged 65 years and older who were consecutively recruited from a geriatric outpatient clinic. All participants provided written informed consent. A total of 156 individuals were enrolled. Exclusion criteria were severe acute medical conditions, inability to communicate, or refusal to participate. For participants with advanced cognitive impairment, psychiatric disorders, or delirium, data were obtained through proxy respondents (family members or caregivers). A total of 12 participants (7.7%) required proxy assistance due to cognitive or communication difficulties. Sociodemographic and clinical characteristics, including age, sex, cohabitation status, educational level, smoking and alcohol use, body mass index (BMI), comorbidities, urinary incontinence, history of falls, and number of medications, were recorded. This study was conducted and reported in accordance with the COSMIN (Consensus-based Standards for the selection of health Measurement INstruments) methodology for studies evaluating measurement properties and the STARD 2015 reporting guideline for diagnostic accuracy studies [16,17].

### 2.2. Human Ethics and Consent to Participate

This study was conducted in accordance with the Declaration of Helsinki and was approved by the Ethics Committee of Bilkent City Hospital (Approval No:1-25-920). All participants provided written informed consent prior to participating in the study.

### 2.3. Sample Size and Power Analysis

An a priori power analysis was conducted using G*Power 3.1.9.7 software to determine the adequacy of the sample size. With a significance level of α = 0.05 and statistical power set at 0.80, the minimum required sample size was calculated as 140 participants. The final sample of 156 participants exceeded this minimum requirement. This sample size was considered sufficient to detect large effect sizes, although the study may not be powered to detect smaller effect sizes.

### 2.4. Reference Standard for Frailty

Frailty status was determined using the Clinical Frailty Scale (CFS) version 2.0. Participants with CFS scores of 1–3 were classified as robust, whereas those with CFS ≥ 4 were classified as frail based on overall clinical condition, functional capacity, and level of independence. This classification was used as the reference standard in all validity analyses [18]. Although no universally accepted gold standard for frailty assessment exists, the CFS was selected as the reference standard because of its extensive validation in geriatric populations, widespread use in routine clinical practice, applicability in outpatient settings, and strong predictive validity for adverse health outcomes in older adults [7]. This multidimensional assessment approach is consistent with the WHO Integrated Care for Older People (ICOPE) framework [19].

The Pictorial Fit–Frail Scale (PFFS), the instrument under validation, is a practical and multidimensional tool that assesses frailty across multiple domains, including mobility, physical activity, balance, cognitive status, social interaction, medication use, mood, and activities of daily living using visual representations [12].

All participants underwent comprehensive geriatric assessment. Functional status was evaluated using the Katz Activities of Daily Living (ADL) Scale [20] and the Lawton–Brody Instrumental Activities of Daily Living (IADL) Scale [21]. Depressive symptoms were screened using the short form of the Geriatric Depression Scale (GDS) [22] and nutritional status was assessed using the Mini Nutritional Assessment—Short Form (MNA-SF) [23]. Cognitive status was evaluated using the Standardized Mini-Mental State Examination (SMMSE) [24,25] based on patient interviews and caregiver reports when necessary. Comorbidity burden was assessed using the Charlson Comorbidity Index, a validated tool that quantifies disease burden and mortality risk by assigning weighted scores to chronic conditions [26].

### 2.5. Translation and Adaptation Process

The Turkish adaptation of the PFFS was conducted in accordance with established international guidelines for cross-cultural adaptation of patient-reported outcome measures.

First, the original PFFS was independently translated into Turkish by two bilingual translators whose native language was Turkish. One translator was a geriatrician familiar with the construct of frailty, while the other was a professional translator without a medical background, ensuring both conceptual and linguistic equivalence.

The two forward translations were compared and synthesized into a single preliminary Turkish version through consensus within the research team.

This version was then back-translated into English by two independent translators whose native language was English and who were blinded to the original instrument. The back translations were reviewed against the original scale to identify inconsistencies or conceptual discrepancies.

### 2.6. Reliability Analysis

The internal consistency of the Turkish version of the PFFS was evaluated using Cronbach’s alpha coefficient.

To assess inter-rater reliability, the PFFS was independently administered by two trained geriatricians in a subgroup of 18 participants, and agreement between raters was evaluated using the intraclass correlation coefficient (ICC) with 95% confidence intervals.

For intra-rater reliability (test–retest reliability), the PFFS was re-administered to the same 18 participants after a 2-week interval by the same evaluator, and ICC values were calculated to assess score stability over time.

An ICC value greater than 0.75 was considered indicative of excellent reliability.

### 2.7. Construct Validity

We hypothesized that the Turkish version of the PFFS would demonstrate strong positive correlations with the Clinical Frailty Scale and adequate diagnostic accuracy for identifying frailty. Construct validity was examined by evaluating the correlation between PFFS total scores and CFS scores using Spearman’s rank correlation coefficient (r).

### 2.8. Statistical Analysis

All statistical analyses were performed using SPSS software 27.0 (IBM Corp., Armonk, NY, USA). Continuous variables were tested for normality using visual inspection of histograms and the Kolmogorov–Smirnov test. Variables with normal distribution were expressed as mean ± standard deviation and compared between robust and frail groups using the independent samples *t*-test. Non-normally distributed continuous variables were expressed as median (25th–75th percentile) and compared using the Mann–Whitney U test. Categorical variables were presented as number and percentage and compared using the chi-square test.

To evaluate the diagnostic performance of the PFFS in identifying frailty, Receiver Operating Characteristic (ROC) curve analysis was performed using frailty classification based on the Clinical Frailty Scale (CFS ≥ 4) as the reference standard. The discriminative ability of the PFFS was quantified using the area under the curve (AUC) with 95% confidence intervals. The optimal cut-off value was determined using the Youden index, calculated as:

J = Sensitivity + Specificity – 1

The cut-off value yielding the maximum Youden index was selected as the optimal threshold for discriminating frail from robust participants. A *p*-value of <0.05 was considered statistically significant.

**Figure 1:**
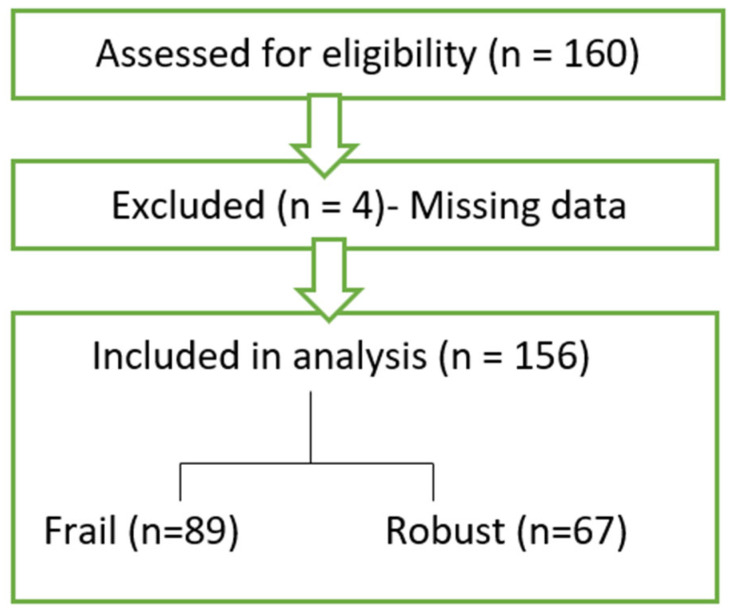
STARD flow diagram of participant recruitment, exclusions, and final analytic sample.

A complete-case analysis was performed. Four participants with missing data were excluded from the final analyses, and all statistical analyses were conducted on the remaining complete dataset. The participant recruitment, exclusions, and final analytic sample are shown in Figure 1. 

## 3. Results

A total of 156 older adults were included in the study, of whom 99 (63.5%) were women. According to the Clinical Frailty Scale, 89 participants (57.1%) were classified as frail and 67 (42.9%) as robust. The mean age of the overall study population was 78.1 ± 8.0 years.

Compared with robust participants, frail individuals were significantly older (80.57 ± 7.99 vs. 74.84 ± 6.82 years, *p* < 0.001) and had a higher comorbidity burden as reflected by higher Charlson Comorbidity Index scores (6 [4–7] vs. 4 [3–6], *p* < 0.001). No significant differences were observed between the groups regarding sex distribution, living arrangements, educational level, or number of medications used (all *p* > 0.05).

Frail participants demonstrated significantly poorer functional status, with lower Katz Activities of Daily Living scores (5 [2–5] vs. 6 [5–6], *p* < 0.001) and lower Lawton–Brody Instrumental Activities of Daily Living scores (4 [1–6] vs. 8 [7–8], *p* < 0.001). Nutritional status was also worse among frail individuals, as indicated by lower MNA-SF scores (11 [11–12] vs. 12 [10–14], *p* < 0.001). In addition, frail participants had lower cognitive performance measured by the SMMSE (25 [18–28] vs. 28 [24–29], *p* < 0.001). Although GDS scores tended to be higher in the frail group, the difference did not reach statistical significance (*p* = 0.115).

As anticipated, frail participants had significantly higher CFS scores than robust participants (5 [4–6] vs. 3 [2–3], *p* < 0.001). Similarly, PFFS scores were significantly higher among frail individuals (17 [12–22] vs. 9 [6–13], *p* < 0.001) (Table 1).

### 3.1. Reliability of the Turkish PFFS

The Turkish version of the PFFS demonstrated high internal consistency, with a Cronbach’s alpha coefficient of 0.838. Inter-rater reliability was excellent, with an intraclass correlation coefficient (ICC) of 0.995 (95% CI: 0.988–0.998). Intra-rater (test–retest) reliability was similarly excellent, yielding an ICC of 0.996 (95% CI: 0.989–0.998).

### 3.2. Construct Validity

The PFFS showed a strong positive correlation with the CFS score (Spearman’s r = 0.760, *p* < 0.001), supporting its construct validity.

### 3.3. ROC Curve Analysis

**Figure 2:**
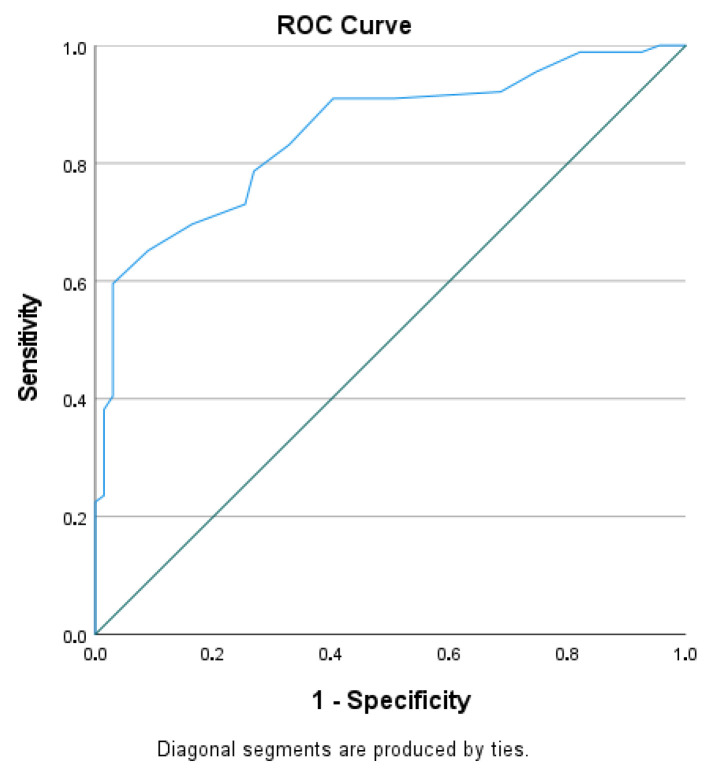
ROC curve of the Turkish Pictorial Fit–Frail Scale (PFSS) for detecting frailty. The PFSS demonstrated good discriminative ability for identifying frailty, with an AUC of 0.851 (95% CI: 0.791–0.910; *p* < 0.001). The optimal cut-off value was ≥14 points, with a sensitivity of 69.7% and a specificity of 83.6%.

ROC analysis demonstrated good discriminative ability of the Turkish PFFS for identifying frailty (AUC = 0.851, 95% CI: 0.791–0.910; *p* < 0.001). The optimal cut-off value was ≥14, yielding a sensitivity of 69.7% (95% CI: 59.0–79.0%), a specificity of 83.6% (95% CI: 72.5–91.5%), and a Youden index of 0.533 (Figure 2).

## 4. Discussion

In this study, the Turkish version of the PFFS demonstrated excellent reliability, supporting its reproducibility across both different raters and repeated assessments. The observed internal consistency indicates that the items assess the construct of frailty coherently without excessive redundancy. These findings are consistent with the original validation study by Theou et al. and subsequent validation studies conducted in different clinical settings, supporting the robustness of the PFFS across diverse populations [12,27].

The strong association between the PFFS and the Clinical Frailty Scale supports the construct validity of the Turkish version. This finding is consistent with previous validation studies reporting good agreement between the PFFS and established frailty instruments, suggesting that the scale captures the multidimensional nature of frailty across different populations [3,4,28].

The Turkish PFFS demonstrated good discriminative ability for identifying frailty. The selected cut-off was based on the Youden index, which provides the best balance between sensitivity and specificity. Although lower thresholds could improve sensitivity, this would be achieved at the expense of reduced specificity and a greater number of false-positive classifications. Conversely, the selected threshold may fail to identify approximately one-third of frail individuals, highlighting that the PFFS should complement rather than replace comprehensive geriatric assessment and clinical judgment, particularly when frailty is clinically suspected. Future studies should investigate whether different cut-off values may be preferable in settings where maximizing sensitivity is the primary objective, such as community-based frailty screening.

The clinical characteristics observed among frail participants are consistent with the multidimensional nature of frailty described in previous studies. The coexistence of functional dependence, cognitive impairment, poor nutritional status, and multimorbidity reinforces the concept that frailty extends beyond physical decline and involves multiple interacting domains [29,30,31]. These findings further support the multidimensional structure of the PFFS.

An important strength of the PFFS is its visual and multidimensional design. Unlike many conventional frailty instruments that rely on performance testing or verbal questionnaires, the PFFS can be completed by patients, caregivers, or healthcare professionals, making it particularly suitable for older adults with communication difficulties, cognitive impairment, or limited health literacy. In addition, its quick application facilitates its use in clinical practice. Previous studies have also shown that visual assessment tools are more understandable and applicable in individuals with different cognitive and communication levels. The PFFS has already demonstrated its applicability and good diagnostic performance in patients with varying levels of cognitive impairment in memory disorder clinics [15]. In addition to its visual format, the PFFS offers a comprehensive multidimensional assessment of frailty. Previous research has suggested that an ideal frailty instrument should evaluate multiple domains, including nutritional status, mobility, physical activity, strength, energy, cognition, mood, and social support. The PFFS incorporates these key dimensions while providing a simpler and more practical alternative to more complex frailty indices. This multidimensional approach may contribute to its construct validity and enhance its applicability across diverse clinical and cultural settings [15,32].

The strengths of this study include the standardized cross-cultural adaptation process, comprehensive psychometric evaluation, inclusion of older adults with varying levels of frailty, and assessment of both inter-rater and test–retest reliability.

This study has several limitations. First, it was conducted in a single center, which may limit the generalizability of the findings. Although the sample size was adequate for validation analyses, larger multicenter studies would further strengthen the evidence supporting the psychometric properties of the scale and provide more robust and stable estimates. In addition, the assessment of participants with severe cognitive impairment was, in some cases, based on information provided by family members or caregivers. While this approach reflects real-world clinical practice, future studies should evaluate the performance of the scale across different patient subgroups and raters to further confirm its applicability. The use of proxy respondents in a small proportion of participants (7.7%) may introduce information bias, as proxy perceptions may not fully reflect participants’ subjective status. However, this reflects real-world clinical practice in cognitively impaired older adults. Future studies should evaluate agreement between patient- and proxy-reported PFFS scores.

Future multicenter studies with larger and more diverse populations are warranted to further confirm these findings and strengthen external validity. In addition, the responsiveness of the Turkish PFFS to longitudinal clinical changes and intervention-related outcomes should be evaluated to further establish its utility in both clinical practice and research settings.

## 5. Conclusions

The Turkish version of the PFFS demonstrated satisfactory validity and excellent reliability for assessing frailty in older adults. Its visual, multidimensional, and easy-to-administer format may facilitate frailty assessment in clinical practice, particularly among individuals with low health literacy or communication difficulties.

## Figures and Tables

**Table 1:** General characteristics of the study population.

	Robust (*n* = 67)	Frail (*n* = 89)	*p*
Age	74.84 ± 6.82	80.57 ± 7.99	**<0.001**
Women Men	41 (61.2%) 26 (38.8%)	58 (65.2%) 31 (34.8%)	0.610
Living alone Living with a family member or caregiver	24 (35.8%) 43 (64.2%)	28 (31.5%) 61 (68.5%)	0.567
Education ≤5 years >5 years	40 (59.7%) 27 (40.3%)	62 (69.7%) 27 (30.3%)	0.195
CCI	4 (3–6)	6 (4–7)	**<0.001**
Number of medications	2 (1–2)	2 (2–2)	0.253
Katz ADL	6 (5–6)	5 (2–5)	**<0.001**
Lawton–Brody IADL	8 (7–8)	4 (1–6)	**<0.001**
GDS	3 (1–5)	4 (1–7)	0.115
MNA-SF	12 (10–14)	11 (11–12)	**<0.001**
CFS	3 (2–3)	5 (4–6)	**<0.001**
SMMSE	28 (24–29)	25 (18–28)	**<0.001**
PFFS	9 (6–13)	17 (12–22)	**<0.001**

CCI: Charlson Comorbidity Index, GDS: Yesavage Geriatric Depression scale, MNA-SF: Mini Nutritional Assessment—Short Form, CFS: Clinical Frailty Scale, SMMSE: Standardized Mini-Mental State Examination, PFFS: Pictorial Fit–Frail Scale. Results are presented as mean ± standard deviation for parametric variables (age); median (25th–75th percentile) for non-parametric variables; and number (percentage) for categorical variables.

## Data Availability

The datasets used and/or analyzed during the current study are available from the corresponding author on reasonable request.
